# Concurrent and Delayed Behavioral and Monoamine Alterations by Excessive Sucrose Intake in Juvenile Mice

**DOI:** 10.3389/fnins.2020.00504

**Published:** 2020-05-19

**Authors:** Won-Hui Choe, Kyung-A Lee, Yukiori Goto, Young-A Lee

**Affiliations:** ^1^Department of Food Science and Nutrition, Daegu Catholic University, Gyeongsan, South Korea; ^2^Primate Research Institute, Kyoto University, Kyoto, Japan

**Keywords:** neurodevelopment, monoamine, reward, stress, anxiety, sucrose

## Abstract

Our daily diet in the modern society has substantially changed from that in the ancient past. Consequently, new disorders associated with such dietary changes have emerged. For instance, excessive intake of compounds, such as sucrose (SUC), has recently been reported to induce pathological neuronal changes in adults, such as food addiction. It is still largely unclear whether and how excessive intake of such nutrients affects neurodevelopment. We investigated changes in behavior and monoamine signaling caused by excessive, semi-chronic intake of SUC and the non-caloric sweetener saccharin (SAC) in juvenile mice, using a battery of behavioral tests and high-performance liquid chromatography. Both SUC and SAC intake induced behavioral alterations such as altered amphetamine responses, sucrose preference, stress response, and anxiety, but did not affect social behavior and cognitive function such as attention in juvenile and adult mice. Moreover, SUC and SAC also altered dopamine and serotonin transmission in mesocorticolimbic regions. Some of these behavioral and neural alterations were triggered by SAC and SUC but others were distinct between the treatments. Moreover, alterations induced in juvenile mice were also different from those observed in adult mice. These results suggest that excessive SUC and SAC intake during the juvenile period may cause concurrent and delayed behavioral and monoamine signaling alterations in juvenile and adult mice, respectively.

## Introduction

Several major components of our diets, such as sugar, fat, and caffeine, have been reported to induce addictive behavior. In modern society, food addiction is becoming a serious social problem, and although it has not yet been classified, food addiction is currently under debate for inclusion into the category of substance use disorder in further diagnostic manuals (Meule and Gearhardt, 2014). The prevalence of food addiction has increased exponentially since the 2000s (Randolph, 1956; Hebebrand et al., 2014; Meule, 2015). Moreover, food addiction could be more prevalent in the presence of other psychiatric disorders (Murphy et al., 2014; Cathelain et al., 2016).

Dopamine (DA) plays a central role in the brain’s reward system. In particular, sucrose (SUC) consumption evokes DA release in the nucleus accumbens (NAcc) in normal mice (Hajnal et al., 2004), whereas dopamine transporter (DAT) knockout mice exhibit decreased SUC preference (Cinque et al., 2018), suggesting that SUC is a robust modulator of the DA system. Moreover, excessive intake of nutrients such as SUC has been demonstrated to cause dysfunction of the DA reward system (Avena et al., 2008; Volkow et al., 2011). Serotonin (5-HT) is monoamine and is also involved in reward mechanisms, which may or may not be mediated through interaction with the DA system (Smolders et al., 2001; Müller and Homberg, 2015).

Accumulating evidence suggests that food addiction involves neural mechanisms that may substantially overlap with those causing drug addiction (Lenoir et al., 2007). Nevertheless, the mechanisms of food addiction have largely remained unclear. Cocaine and nicotine addiction induce structural alterations of neurons in the prefrontal cortex (PFC) and the NAcc (Robinson and Kolb, 2004), which could be associated with impairments of cognitive and affective function (Quello et al., 2005; Gould, 2010). However, whether food addiction also involves similar neuronal and behavioral alterations is mostly unexplored. In addition, since drug addiction is primarily a problem of adolescent and adult subjects, neurodevelopmental aspects have rarely been considered. Given that addictive diets such as SUC are consumed from a very early developmental stage, the impact of exposing humans to such addictive diets at early neurodevelopment may play a critical role.

Excessive intake of both caloric sweeteners, such as SUC, and non-caloric artificial sweeteners, such as saccharin (SAC) and aspartame, has been reported to induce addiction (Murray et al., 2016). Excessive aspartame consumption has been reported to cause irritable mood, depression, and to impair spatial memory (Lindseth et al., 2014). Long-term consumption of artificial sweeteners also disrupts passive avoidance learning memory (Erbaş et al., 2018). These studies raise the question of whether behavioral and neural changes associated with food addiction are induced by excessive intake of caloric nutrients or just by an excessive rewarding (sweet taste) experience.

In this study, we investigated the effects of excessive intake of SUC and SAC during the early neurodevelopmental period on cognitive, affective, and social functions and on DA/5-HT signaling in mesocorticolibmic brain regions of mice. We hypothesized that excessive intake of both SAC and SUC causes persistent behavioral and monoamine signaling alterations until adulthood and that such alterations may partly differ between SAC and SUC.

## Materials and Methods

### Animals

All animal experiments were conducted in accordance with the Research Ethics Policy of the Korean Association of Laboratory Animal Science and were approved by the Institutional Animal Care and Use Committee of Daegu Catholic University. Pregnant female ICR mice at gestational day 14 were individually housed. Male pups were weaned from dams at postnatal day (PD) 21 and housed in cages of four to five mice per cage for subsequent experiments. To avoid (to minimize) litter effects, a number of mice born from each dam was limited to 3 offspring. Moreover, these littermates were separated at birth and raised by different dams.

### SUC and SAC Treatments in Juvenile Mice

**FIGURE 1:**
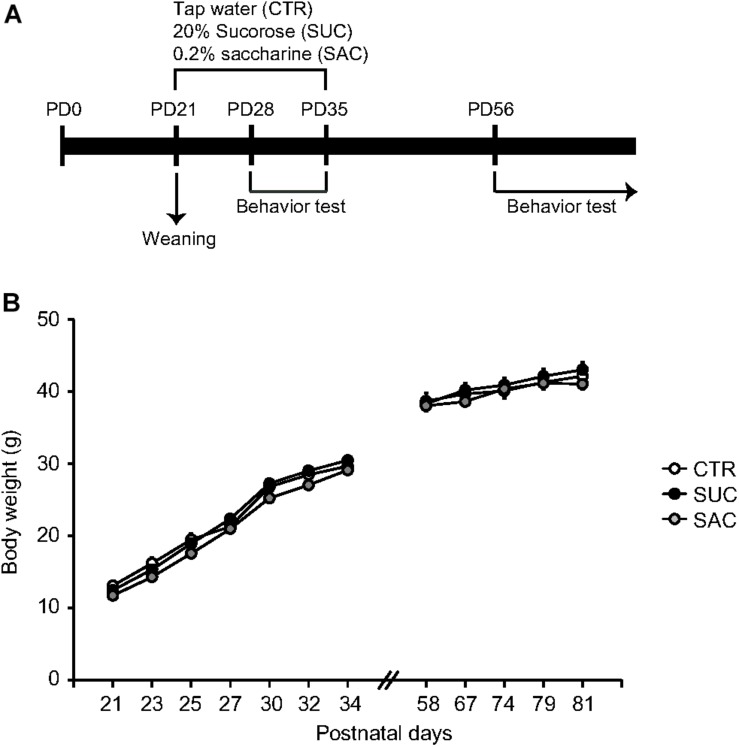
Experimental schedule and body weight gain. **(A)** Schematic diagram illustrating the timeframe of experiments. **(B)** Graph showing body weight gain of animals with vehicle (CTR), 20% sucrose (SUC), and 0.2% saccharine (SAC) treatments. Error bars indicate SEM.

Starting at PD21, animals were assigned into three groups. The first group of animals with control (CTR) treatment received only tap water. Animals in the second and third groups received 20% SUC or 0.2% SAC solution, respectively. These solutions were available *ad libitum* until PD35. All animals received tap water after PD36 (Figure 1A). The concentrations of SAC and SUC solutions were based on previous studies (Feijó et al., 2013; Bissonnette et al., 2017). SUC and SAC treatments during the juvenile period resulted in no changes in body weight gain compared to CTR treatment (Figure 1B).

### Behavioral Tests

All behavioral tests were conducted during the juvenile period (PD28–35; Figure 1A) and adulthood (PD56 or older; Figure 1A).

#### Locomotion Test With Amphetamine Treatment

The DA response was examined with locomotion tests after amphetamine (AMP) administration (Salahpour et al., 2008). Animals were placed in an open-field chamber and the locomotor distance that animals traveled during 20 min was measured, either after AMP or saline administration. AMP at doses of 1, 2, and 3 mg/kg dissolved in 0.1 mL saline and the equivalent volume of saline was given intraperitoneal repeatedly to the same animals once per day over 4 days. These injections were administered 10 min before the animal was subjected to the locomotion test.

#### SUC Preference Test

A SUC preference test was conducted to examine whether SUC and SAC treatments affected reward sensitivity (Hajnal et al., 2004). During this test, animals were individually housed in cages with two water bottles: one with tap water and the other with SUC solution. Different concentrations of SUC (0, 0.25, 0.5, 1.0, 2.0, and 5.0%) were offered to the animals on each day. The amount of SUC solution and tap water intake was measured. SUC solution and tap water were offered for 16 h (17:00–09:00) at each SUC concentration condition. The preference to SUC over tap water was expressed as the preference ratio, which was the amount of SUC solution consumption compared to tap water consumption.

#### Forced Swimming Test

A forced swimming test was conducted to examine responsiveness to stress (Yankelevitch-Yahav et al., 2015). We utilized the forced swimming test over other test such as Morris water maze test, as the aim of conducting this study was not focused on spatial learning and memory, but sorely stress response. A glass cylinder (50 cm height × 11 cm diameter) was filled with tap water at 23 ± 1°C (35 cm from the bottom). Animals were placed into the glass-cylinder for 10 min and the total duration of immobility in the water was measured.

#### Elevated Plus Maze Test

An elevated plus maze test was conducted to examine anxiety of animals (Walf and Frye, 2007). The elevated plus maze consisted of two diagonal arms without side walls (open arms) and two arms with walls perpendicular to the open arms (enclosed arms). Animals were placed in the center area of the maze and allowed to freely enter the arms for 10 min. Duration and number of entries in the open and enclosed arms were measured.

#### Object Exploration Test

An object exploration test that we have developed previously (Kim et al., 2018) was conducted to examine attention. In this test, four objects with different features were placed in an open-field chamber. Animals were then placed in the chamber and allowed to explore these objects freely for 10 min. Their exploratory behaviors (touching, sniffing, and riding) over the objects were considered as animals paid attention to the objects, allowing us to measure the time spent to explore these objects.

#### Social Interaction Test

A social interaction test was conducted to examine sociality of animals (Innos et al., 2011). In this test, an animal was placed in the open-field chamber with another, age-matched, unfamiliar animal. Duration and frequency of demonstrated social behaviors such as sniffing and chasing were measured for 10 min. In cases where one of the animals showed aggressive attacks, such as biting and jumping, the experiment was stopped, and a new experiment was conducted on another day.

### High-Performance Liquid Chromatography

High-performance liquid chromatography (HPLC) was conducted to investigate tissue concentrations of DA and 5-HT and their metabolites in mesocorticolimbic brain regions. Animals were euthanized by administration of an overdose of sodium pentobarbital [100 mg/kg, intraperitoneal (i.p.)] and zoletil (60 mg/kg, i.p.), followed by decapitation. The brains were removed and cut into coronal sections according to the stereotaxic coordinates of the atlas (Franklin and Paxinos, 2008). Then tissue samples from the medial PFC, the dorsal striatum (dSTR), the NAcc, the basolateral and lateral nuclei of the amygdala (AMY), the dorsal part of the hippocampus (HPC), and the ventral tegmental area (VTA) were obtained using disposable biopsy punch (diameter 1.0 mm, Kai Industries, Co., Ltd., Japan). The tissue samples were processed as described previously (Lee et al., 2018; Jeon et al., 2019). Quantification of DA, 5-HT, 3,4-dihydroxyphenylacetic acid (DOPAC), homovanillic acid (HVA), and 5-hydroxyindoleacetic acid (5-HIAA) was conducted according to the formula *(ISO_*std*_/TA_*std*_) x (TA_*spl*_/ISO_*spl*_) x A x (1/B)*, where *ISO*_*std*_ and *TA*_*std*_ are areas under the curves of the peaks in chromatograms for isoproterenol (ISO) added in standard and sample solutions as the internal standard marker) and a target substance in the standard solution, respectively. *ISO*_*spl*_ and *TA*_*spl*_ are areas under the curves of the peaks for ISO and target substances in the sample solution, respectively. *‘A’* is the amount of ISO added to sample solution. *‘B’* is the amount of tissue proteins in sample solution.

### Immunostaining

Fluorescent immunostaining was conducted to examine DAT and serotonin transporter (SERT) expression in mesocorticolimbic regions. An overdose of sodium pentobarbital (100 mg/kg, i.p.) and zoletil (60 mg/kg, i.p.) was administered to animals and once no response to tail pinches was confirmed, the abdomen was surgically opened. Then, transcardiac perfusion of phosphate buffer saline (PBS) and 4% paraformaldehyde was conducted to remove the blood and fix the brain. After perfusion, the brains were removed and immersed in 4% paraformaldehyde, followed by 30% SUC solution in PBS for anti-freezing protection. After these procedures, the brains were cut into slices of 30 μm thickness using a microtome and placed on gelatin-coated slide glasses. The slides were washed with 0.3% Triton X in PBS and incubated with DAT (Catalog #: sc14002, Rabbit polyclonal, dilution at 1:300, Santa Cruz Biotechnology) and SERT (Catalog #: sc33724, Mouse monoclonal, dilution at 1:300, Santa Cruz Biotechnology) antibodies with 10% normal goat serum diluted in 0.3% Triton X in PBS at 4°C for 24 h. Afterward, the slides were incubated with Alexa Fluor 488 goat anti-mouse IgG (H+L) (Catalog #: A-11001, dilution at 1:300, Thermo Fisher Scientific) and Alexa Fluor 594 goat anti-rabbit IgG (H+L) (Catalog #: A-11012, dilution at 1:300, Thermo Fisher Scientific) secondary antibodies for DAT and SERT, respectively, diluted in 0.3% Triton X in PBS at room temperature for 2 h. The slides were covered with cover glasses with mounting medium. Fluorescence intensity was captured with the fluorescence microscope (DM2500, Leica), and images were analyzed using ImageJ (version 1.51j8). The images were converted into 8-bit grayscale, and then intensity was quantified under the threshold set equally applied to all images. As a control for these specific expressions, shadow effects of primary antibodies were also examined, and illustrated in Supplementary Figure S1.

### Data Analysis

All data are expressed as means ± SEM *p* < 0.05 was considered statistically significant different. Statistical analysis for comparisons among CTR, SUC, and SAC groups was conducted using analysis of variance (ANOVA), with *post hoc* Turkey test for pair-wise comparisons.

## Results

### Alterations of the AMP Response

**FIGURE 2:**
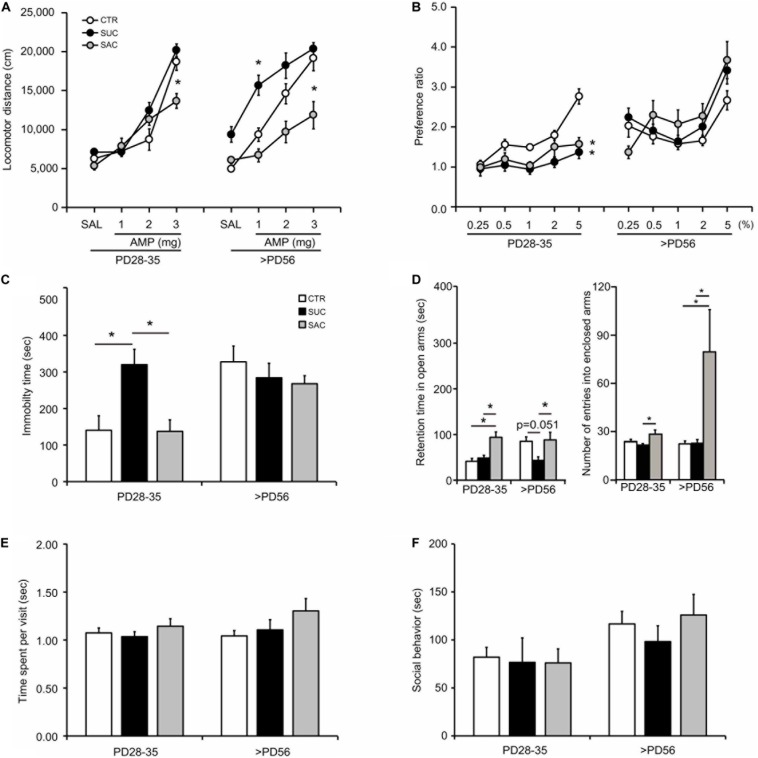
Behavioral alterations in animals subjected to sucrose and saccharine treatments. **(A)** Graph showing amphetamine (AMP) modulation of locomotion in juvenile (PD28–35) and adult (>PD56) animals. **(B)** Graph showing sucrose preference expressed as the ratio of sucrose solution intake over tap water intake in the sucrose preference test. **(C)** Graph showing the duration of immobility in the forced swimming test. **(D)** Graph showing the time spent in the open arms (left) and the number of entries into the enclosed arms (right) in the elevated plus maze. **(E)** Graph showing the time spent for object exploration per visit in the object exploration test. **(F)** Graph showing the time spent on social interaction with a mate in the social interaction test. *p < 0.05.

Whether juvenile SUC and SAC intake affects the monoamine system was examined via AMP modulation of locomotion in the open field chamber (Figure 2A). When tested in juvenile mice (PD28–35), CTR, SUC, and SAC animals exhibited no difference in locomotor distance with SAL and 1 and 2 mg/kg AMP administration. However, locomotor distance in SAC animals with 3 mg/kg AMP administration was lower than in CTR animals (two-way ANOVA with repeated measures; *F*_2_,_180_ = 8.905, *p* < 0.001 for groups; *F*_3_,_180_ = 106.423, *p* < 0.001 for AMP; *F*_6_,_180_ = 5.353, *p* < 0.001 for interaction; *post hoc* Tukey test; *p* = 0.021, CTR vs. SAC). In adulthood (PD56 or older), shifts of the dose-response curves were observed, for example of the AMP response in SUC animals toward higher AMP sensitivity along with a significantly higher locomotor distance than in CTR animals with 1 mg/kg AMP (*F*_2_,_140_ = 39.132, *p* < 0.001 for groups; *F*_3_,_140_ = 42.540, *p* < 0.001 for AMP; *F*_6_,_140_ = 2.897, *p* = 0.011 for interaction; *p* = 0.010, CTR vs. SUC). In contrast, the AMP response in SAC animals was shifted toward more blunted sensitivity along with a significantly lower locomotor distance than in CTR animals with 3 mg/kg AMP (*p* = 0.021, SAC vs. CTR). These results suggest that both SAC and SUC treatments may cause alterations in AMP responses that persist up until adulthood. However, these alterations differed between SAC and SUC.

### Alterations of SUC Preference

Juvenile CTR animals exhibited higher SUC preference over increasing SUC concentrations (Figure 2B). Two-way ANOVA with repeated measures revealed significant differences in the interaction between groups and SUC concentrations (*F*_2_,_148_ = 11.484, *p* < 0.001 for groups; *F*_4_,_148_ = 18.526, *p* < 0.001 for SUC concentrations; *F*_8_,_148_ = 3.578, *p* < 0.001 for interaction). *Post hoc* analysis revealed that SUC preference in CTR animals was higher at 2% (*p* = 0.018) and 5% (*p* < 0.001) SUC than at 0.25%. This increase was not observed in SUC and SAC mice, resulting in significantly lower SUC preference than in CTR mice at 5% SUC (*p* = 0.006, CTR vs. SUC; *p* = 0.025, CTR vs. SAC). In contrast, no difference in SUC preference was observed between adult CTR, SUC, and SAC animals. Some of the mice were already sucrose-exposed prior to the sucrose test, such that a novelty aspect of the sucrose might be unequal among the groups. However, as shown in Figure 2B, our results indicate that differences were prominent at higher doses of sucrose solution, which were the conditions examined after animals had already experienced lower doses. Thus, such difference suggest that a novelty might be less likely involved, although the possibility that a novelty to the sucrose was still influencing its preference cannot be excluded. These results suggest that both SUC and SAC treatments may cause a similar, transient SUC preference change in juvenile mice.

### Alterations of Stress Response

The forced swimming test was conducted to evaluate stress responses (Figure 2C). In juvenile mice, SUC animals exhibited longer immobility than CTR and SAC animals (one-way ANOVA; *F*_2_,_25_ = 7.825, *p* = 0.002; *p* = 0.017, CTR vs. SUC; *p* = 0.004, SUC vs. SAC). In contrast, no difference was observed in adult CTR, SUC, and SAC animals. These results suggest that SUC, but not SAC, intake may cause a transient alteration of stress response in juvenile mice.

### Alterations of Anxiety

The elevated plus maze test was conducted to evaluate anxiety (Figure 2D). Juvenile SAC animals stayed in the open arms longer than CTR and SUC animals (*F*_2_,_39_ = 9.604, *p* < 0.001; *p* = 0.004, CTR vs. SAC; *p* = 0.001, SUC vs. SAC). Moreover, juvenile SAC animals entered into the enclosed more often than and SUC (*F*_2_,_39_ = 5.189, *p* = 0.010; *p* = 0.007, SUC vs. SAC). When tested in adulthood, SUC animals spent less time in the open arms than SAC animals (*F*_2_,_35_ = 4.410, *p* = 0.020; *p* = 0.030, SUC vs. SAC; *p* = 0.051, CTR vs. SUC). Moreover, SAC animals entered into the enclosed arm more often than SUC and CTR animals (*F*_2_,_35_ = 4.515, *p* = 0.018; *p* = 0.037, CTR vs. SAC; *p* = 0.034, SUC vs. SAC). These results suggest that both SAC and SUC treatments may cause alterations of anxiety but in a distinct manner across ages.

### No Alteration of Attention and Social Behavior

In the object exploration test to examine attention (Figure 2E) and the social interaction test (Figure 2F), no difference in time spent on object exploration per visit and time spent on social interaction was observed in juvenile and adult CTR, SUC, and SAC animals.

### Alterations of DA and 5-HT

To understand the neural mechanisms that may be associated with the observed behavioral alterations, changes in DA and 5-HT levels in the PFC, dSTR, NAcc, HPC, AMY, and VTA of juvenile and adult animals were examined.

**FIGURE 3:**
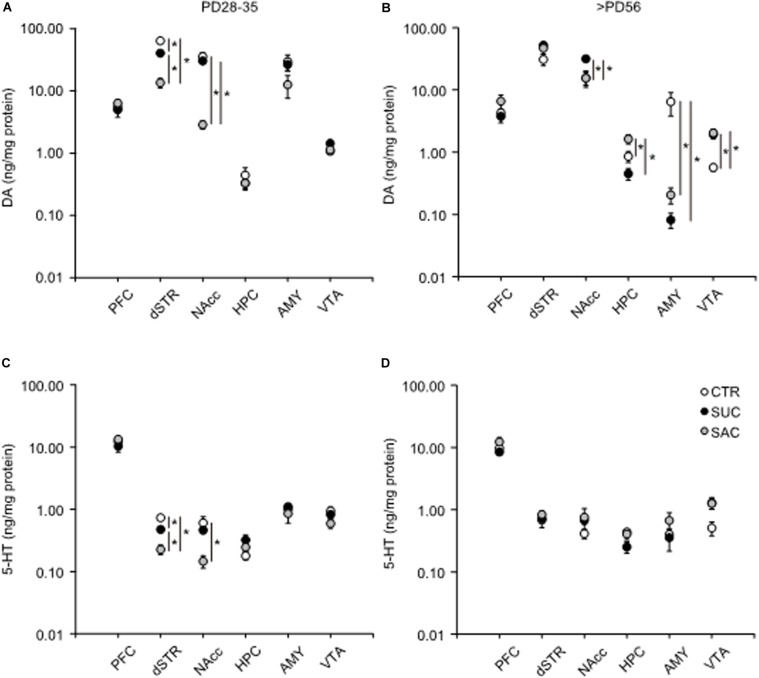
Alterations of tissue DA and 5-HT concentrations in mesocorticolimbic regions of animals subjected to sucrose and saccharine treatments. Graphs showing tissue DA concentrations in the PFC, dSTR, NAcc, HPC, AMY, and VTA of juvenile **(A)** and adult **(B)** animals. Graphs similar to a and b but showing tissue 5-HT concentrations in juvenile **(C)** and adult **(D)** animals. *p < 0.05.

In juvenile mice, DA levels were lower in the dSTR of SUC and SAC animals than of CTR animals (*F*_2_,_15_ = 73.415, *p* < 0.001; *p* < 0.001, CTR vs. SUC; *p* < 0.001, CTR vs. SAC; *p* < 0.001, SUC vs. SAC; Figure 3A). In the NAcc, the DA concentration was also lower in SAC animals than in CTR and SUC animals (*F*_2_,_15_ = 23.573, *p* < 0.001; *p* < 0.001, CTR vs. SAC; *p* < 0.001, SUC vs. SAC; Figure 3A). In addition, the 5-HT concentration in the dSTR of SUC and SAC animals was lower than in CTR animals (*F*_2_,_15_ = 32.473, *p* < 0.001 for groups; *p* = 0.003, CTR vs. SUC; *p* < 0.001, CTR vs. SAC; *p* = 0.003, SUC vs. SAC; Figure 3C). The 5-HT concentration in the NAcc was also lower in SAC animals than in CTR animals (*F*_2_,_15_ = 5.750, *p* = 0.014 for groups; *p* = 0.013, CTR vs. SAC, Figure 3C).

In adulthood, DA was higher in the NAcc of SUC animals than in CTR (*F*_2_,_15_ = 6.130, *p* = 0.011; *p* = 0.022) and SAC (*p* = 0.021) animals (Figure 3B). In the HPC, DA in SAC animals was higher than in CTR (*F*_2_,_15_ = 9.430, *p* = 0.002; *p* = 0.034) and SUC (*p* = 0.002) animals (Figure 3B). In the AMY, SUC and SAC animals exhibited lower DA than CTR animals (*F*_2_,_15_ = 5.726, *p* = 0.014; *p* = 0.025, CTR vs. SUC; *p* = 0.028, CTR vs. SAC; Figure 3B), whereas this was opposite in the VTA. DA in SUC and SAC animals was higher than in CTR animals (*F*_2_,_15_ = 11.616, *p* < 0.001; *p* = 0.003, CTR vs. SUC; *p* = 0.002, CTR vs. SAC, Figure 3B). In contrast, 5-HT did not differ between groups, except the VTA where an overall group difference, but no pair-wise difference, was observed (*F*_2_,_15_ = 3.802, *p* = 0.046 in the VTA; Figure 3D).

**FIGURE 4:**
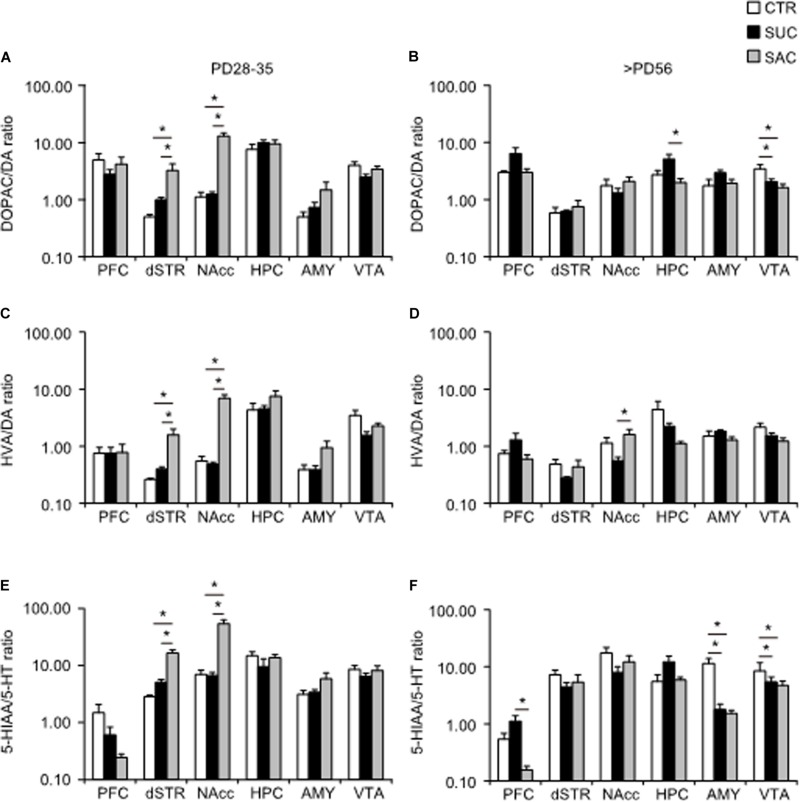
Alterations of ratios between DA/5-HT and their metabolites in mesocorticolimbic regions of animals subjected to sucrose and saccharine treatments. Graphs showing the DOPAC/DA ratio in the PFC, dSTR, vSTR, HPC, AMY, and VTA of juvenile **(A)** and adult **(B)** animals. Graphs similar to a and b but showing HVA/DA ratio in juvenile **(C)** and adult **(D)** animals. Graphs similar to a and b but showing 5-HIAA/5-HT ratio in juvenile **(E)** and adult **(F)** animals. *p < 0.05.

DA and 5-HT metabolisms were further analyzed and the ratio of DOPAC/DA, HVA/DA and HIAA/5-HT, which are thought to reflect synaptic release of DA and 5-HT (Best et al., 2010; Meiser et al., 2013), were examined. In juvenile mice, the DOAPC/DA ratio was higher in the dSTR (*F*_2_,_15_ = 6.924, *p* = 0.007; *p* = 0.009, CTR vs. SAC; *p* = 0.030, SUC vs. SAC) and the NAcc (*F*_2_,_15_ = 43.310, *p* < 0.001; *p* < 0.001, CTR vs. SAC; *p* < 0.001, SUC vs. SAC) of SAC animals than in CTR and SUC animals (Figure 4A). Consistent with such alterations, the HVA/DA ratio was also higher in the dSTR (*F*_2_,_15_ = 8.944, *p* = 0.003; *p* = 0.004, CTR vs. SAC; *p* = 0.010, SUC vs. SAC) and the NAcc (*F*_2_,_15_ = 36.042, *p* < 0.001; *p* < 0.001, CTR vs. SAC, *p* < 0.001, SUC vs. SAC) of SAC animals than in CTR and SUC animals (Figure 4C). Similarly, the 5-HIAA/5-HT ratio was higher in the dSTR (*F*_2_,_15_ = 30.347, *p* < 0.001; *p* < 0.001 for CTR vs. SAC, *p* < 0.001 for SUC vs. SAC) and the NAcc (*F*_2_,_15_ = 28.199, *p* < 0.001; *p* < 0.001 for CTR vs. SAC, *p* < 0.001 for SUC vs. SAC) of SAC animals than in CTR and SUC animals (Figure 4E).

In adulthood, an overall group difference, but no pair-wise difference, of the DOPAC/DA ratio was found in the PFC (*F*_2_,_15_ = 3.785, *p* = 0.047; Figure 4B). In the HPC, the DOPAC/DA ratio was higher in SUC animals than in SAC but not CTR animals (*F*_2_,_15_ = 5.916, *p* = 0.013; *p* = 0.013, SUC vs. SAC; Figure 4B). The DOPAC/DA ratio in the VTA of SUC and SAC animals was significantly lower than in CTR animals (*F*_2_,_15_ = 29.069, *p* < 0.001; *p* < 0.001, CTR vs. SUC; *p* < 0.001, CTR vs. SAC; Figure 4B). The HVA/DA ratio in the NAcc of SUC animals was also lower than in SAC but not CTR animals (*p* = 0.048; Figure 4D). The 5-HIAA/5-HT ratio in the AMY (*F*_2_,_15_ = 16.237, *p* < 0.001; *p* = 0.001, CTR vs. SUC; *p* = 0.001, CTR vs. SAC) and the VTA (*F*_2_,_15_ = 5.280, *p* = 0.018; *p* = 0.041, CTR vs. SUC; *p* = 0.027, CTR vs. SAC) of both SUC and SAC animals was lower than in CTR animals (Figure 4F), whereas the 5-HIAA/5-HT ratio in the PFC of SAC animals was lower than in SUC but not CTR animals (*F*_2_,_15_ = 6.721, *p* = 0.008; *p* = 0.007; Figure 4F).

These results suggest that excessive juvenile SUC and SAC intake may induce distinct patterns of immediate and persistent alterations in mesocorticolimbic DA and 5-HT transmission.

### Alterations of DAT and SERT Expression

Alterations of tissue DA and 5-HT levels and their metabolites suggested that synaptic DA and 5-HT release mechanisms may be affected by excessive SUC and SAC intake in juvenile mice. Thus, we also investigated DAT and SERT expression levels in mesocorticolimbic regions of juvenile and adult animals.

**FIGURE 5:**
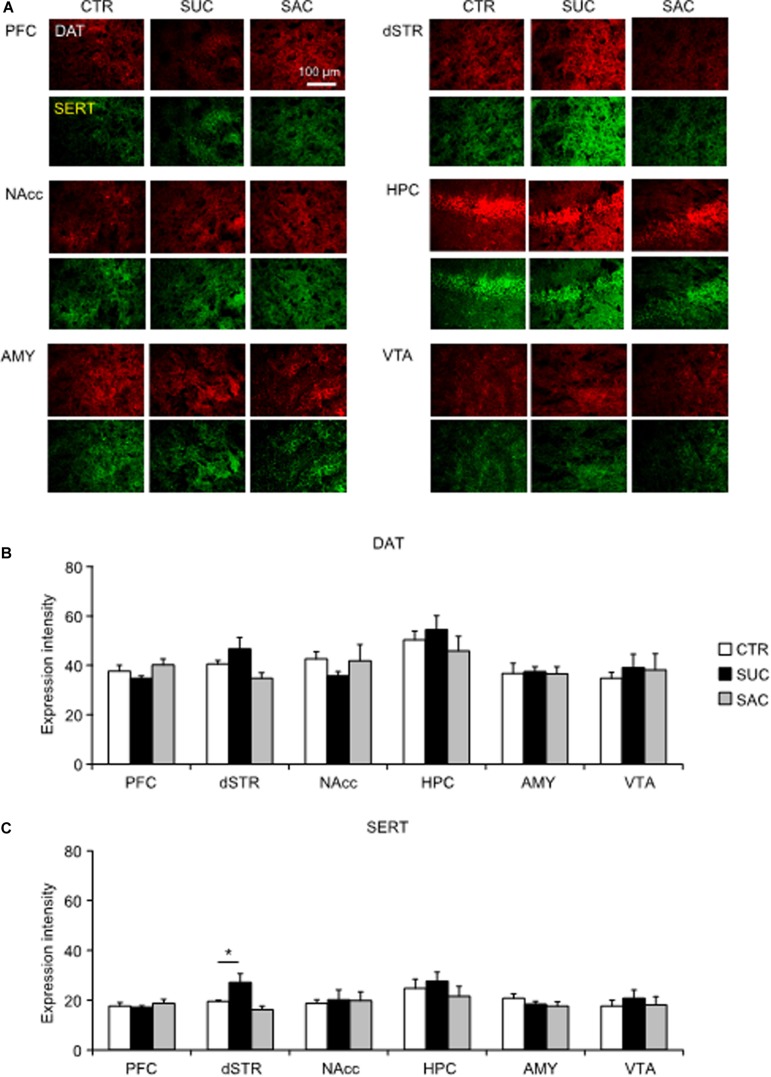
Alterations of dopamine and serotonin transporter expression levels in mesocorticolimbic regions of juvenile animals subjected to sucrose and saccharine treatments. **(A)** Representative images of DAT (red) and SERT (green) expression levels in mesocorticolimbic regions of juvenile animals. Graph showing fluorescence intensities of DAT **(B)** and SERT **(C)** expression in juvenile animals. *p < 0.05.

In juvenile mice, DAT and SERT expression of SUC and SAC animals did not differ from those in CTR animals (Figures 5A–C). The only difference was a higher SERT expression in the dSTR of SUC animals compared to CTR animals (*F*_2_,_13_ = 5.410, *p* = 0.020; *p* = 0.019; Figure 5C).

**FIGURE 6:**
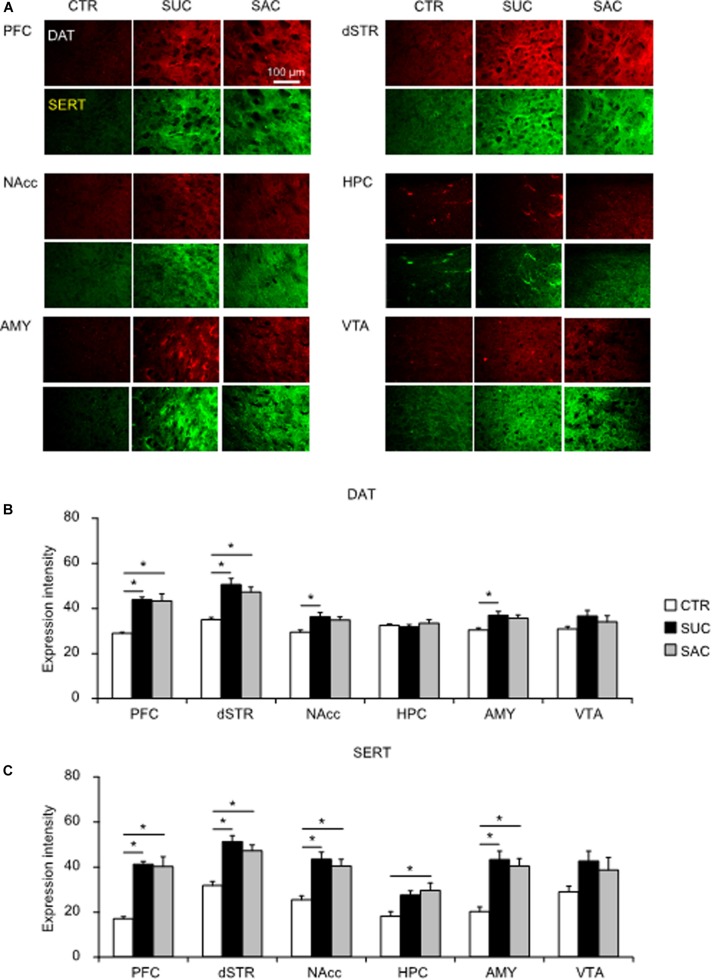
Alterations of dopamine and serotonin transporter expression levels in mesocorticolimbic regions of adult animals subjected to sucrose and saccharine treatments. **(A)** Representative images of DAT (red) and SERT (green) expression in mesocorticolimbic regions of adult animals. Graph showing fluorescence intensities of DAT **(B)** and SERT **(C)** expression in adult animals. *p < 0.05.

In adulthood, significant DAT and SERT expression changes were observed (Figures 6A–C). In the PFC and the dSTR, both SUC and SAC animals exhibited significantly higher DAT expression levels than CTR animals (*F*_2_,_15_ = 12.211, *p* < 0.001 for the PFC; *p* = 0.002 for CTR vs. SUC, *p* = 0.002 for CTR vs. SAC; *F*_2_,_15_ = 11.682, *p* = 0.001 for the dSTR; *p* = 0.001 for CTR vs. SUC, *p* = 0.005 for CTR vs. SAC; Figure 6B). In the NAcc (*F*_2_,_15_ = 5.303, *p* = 0.018; *p* = 0.018 for SUC vs. CTR) and the AMY (*F*_2_,_14_ = 5.000, *p* = 0.023; *p* = 0.023 for SUC vs. CTR), DAT expression levels were higher in SUC animals than in CTR animals (Figure 6B). Group differences of SERT expression levels were observed in all but the VTA (*F*_2_,_15_ = 16.893, *p* < 0.001 for the PFC; *F*_2_,_15_ = 15.570, *p* < 0.001 for the dSTR; *F*_2_,_15_ = 10.323, *p* = 0.002 for the NAcc; *F*_2_,_14_ = 5.367, *p* = 0.019 for the HPC; *F*_2_,_14_ = 13.475, *p* = 0.001 for the AMY; Figure 6C). In these areas, SERT expression was higher in SUC and SAC animals than in CTR animals (*p* < 0.001 for CTR vs. SUC, *p* < 0.001 for CTR vs. SAC in the PFC; *p* < 0.001 for CTR vs. SUC, *p* = 0.002 for CTR vs. SAC in the dSTR; *p* = 0.002 for CTR vs. SUC, *p* = 0.006 for CTR vs. SAC in the NAcc; *p* = 0.001 for CTR vs. SUC, *p* = 0.002 for CTR vs. SAC in the AMY; Figure 6C), although in the HPC, SERT expression was only higher in SAC animals compared to CTR animals (*p* = 0.020 for CTR vs. SAC in the HPC; Figure 6C).

These results suggest that excessive SUC and SAC intake may induce distinct and delayed alterations of DAT and SERT expression levels in mesocorticolimbic regions in adulthood.

## Discussion

In this study, we found that excessive SUC and SAC intake during the juvenile period in rodents induced behavioral and neural alterations, some of which were already apparent in juvenile mice while others became apparent when the animals reached adulthood. The immediate alterations in juvenile mice and delayed alterations in adult mice were different. Moreover, some of the alterations induced by SUC and SAC treatments were common while others differed between SUC and SAC.

This study had two aims. First, we examined how excessive SUC and SAC intake during the juvenile period affected behavioral and neural alterations during development and whether excessive SUC and SAC intake affected behaviors and neural mechanisms not only in juvenile but also in adult animals. Previous studies in adult animals have shown that excessive SUC intake abnormally increases DA release in the NAcc, which is more prominent in the core than the shell regions. Such alteration is similar to that caused by excessive ethanol intake (Kranz et al., 2010; Bassareo et al., 2015). Excessive SUC intake also promotes abnormal 5-HT release in the HPC (Smolders et al., 2001). However, consistent with studies in adult animals, we found that excessive SUC and SAC intake in juvenile mice also altered DA and 5-HT transmission and associated behaviors not only in juvenile but also in adult animals (Supplementary Tables S1–S3). On the other hand, SUC preference was decreased both in SUC and SAC animals during the juvenile period, but not adulthood. Moreover, juvenile but not adult SUC animals exhibited greater vulnerability to stress, suggesting that some behavioral alterations were induced immediately and transiently. In contrast, alterations in AMP modulation of locomotion were observed both in juvenile and adult mice. Importantly, such alterations observed both in juvenile and adult mice were not identical, suggesting that the alterations observed in adulthood are not merely a consequence of excessive SUC and SAC intake during the juvenile period, but become apparent by interaction between juvenile alterations and maturation during adolescence. Consistent with these behavioral alterations, DAT and SERT expression levels in mesocorticolimbic regions were not changed in juvenile SUC and SAC animals, whereas these molecules were significantly up-regulated in several mesocorticolimbic regions of adult animals. In this regard, it is interesting to note that DAT and SERT expression levels were not modulated by SAC and SUC in juvenile animals. Moreover, in adult animals, DAT and SERT expression levels were altered mostly in the regions where tissue DA and 5-HT concentrations and their metabolites were not altered. In contrast, in other regions where DAT and SERT expression levels were not altered, alterations of tissue DA and 5-HT concentrations and their metabolites were observed. These results suggest that up-regulation of DAT and SERT expression in adult animals that had excessive SUC and SAC intake during the juvenile period could be a compensatory action of altered DA and 5-HT transmission.

Several studies have established the role of the intrauterine environment and immediate postnatal period in inducing central DA deficiencies in obesity-prone animals (Geiger et al., 2008). Indeed, the effects of SUC and SAC exposure during an early developmental phase like gestation and weaning would yield substantial impacts on the monoamine systems. In contrast, alterations of monoamine systems observed with the effects of SUC and SAC exposure during the juvenile period were less robust. However, we investigated the effects during the juvenile period, since this study was particularly motivated to investigate the effects of SUC and SAC intakes during the juvenile period, in order to find an insight for the effects of excessive intakes in kids at school ages as human cases, in which excessive intakes of SUC and SAC have often been reported problematic and deteriorating.

Accumulating evidence also suggests that obesity induces specific patterns of DA changes in the STR of both rodents and humans. In rats, obesity has been demonstrated to be associated with decrease of extracellular DA level, concurrent with decreased DAT and D2 receptor expression in the NAcc (Geiger et al., 2008, 2009). Consistent with it, a human study has shown decreased D2 receptor availability in the STR of obese people (Wang et al., 2001). Since we did not examine extracellular DA level or D2 receptor expression, it remains largely unclear how the results are similar or different with those previous findings; however, it appears that the effects of juvenile SAC and SUC intakes could be quite distinct, as tissue DA concentration was found higher in the NAcc, along with higher DAT expression in adult mice exposed to juvenile SUC intake. One possible explanation for such discrepancy is that the effects of SUC and SAC intake are not sorely determined by high caloric intake.

The second aim of our study was to compare the effects of SUC and SAC, for which we directly compared behavioral and neural alterations caused by excessive SUC and SAC intake during the juvenile period with the identical administration procedure. A previous study has shown activation of the striatum with caloric drink but not with non-caloric drink consumption (Smeets et al., 2011). In another study, both SUC and non-caloric artificial sucralose have been reported to similarly activate the primary gustatory cortex. However, SUC, but not sucralose, activates several additional brain regions, such as the anterior insula, the frontal operculum, the striatum, and the anterior cingulate (Burke et al., 2018). In this study, we found that some behavioral and neural alterations were common but we also found differences between the treatments (Supplementary Tables S1–S3). In particular, adult SUC animals exhibited higher sensitivity to AMP, whereas the AMP response was blunted in adult SAC animals. Moreover, juvenile, SAC, but not SUC, animals were less anxious. In adulthood, SUC, but not SAC, animals were more anxious. Juvenile SUC, but not SAC, animals also exhibited greater vulnerability to stress. Consistent with these behavioral alterations, some, but not all, DA and 5-HT alterations were also distinct between SUC and SAC intake. The ratio between DA/5-HT and their metabolites has been defined as the rates of DA and 5-HT turnovers (Nissbrandt and Carlsson, 1987). Studies have shown that cocaine self-administration increases DA turnover in the dSTR and NAcc (Kimmel et al., 2003; Smith et al., 2003). Lower tissue DA and 5-HT concentrations and higher turnovers of them were observed in the dSTR of both juvenile SUC and SAC animals. In addition, lower tissue DA and 5-HT concentrations and higher turnovers of them in the NAcc were also found in juvenile SAC, but not SUC, animals. Such differences in the NAcc may contribute to behavioral alterations observed in juvenile SAC, but not SUC, animals, such as the lower AMP response at the high dose, and the lower anxiety in SAC animals. In adulthood, distinct alterations between SUC and SAC animals were also observed in the NAcc, which may contribute to behavioral alterations observed in adult SUC and SAC animals, such as the higher anxiety in SUC animals and the augmented and blunted AMP responses in SUC and SAC animals, respectively. SAC is a non-caloric, whereas SUC is caloric, sweeteners. Thus, if DA and 5-HT alterations were induced by only repeated exposure to the sweet taste, alterations of DA and 5-HT concentrations and turnovers would not be different between SUC and SAC conditions. However, SUC animals exhibited some distinct behavioral and neural alterations from those in SAC animals, suggesting that alterations induced by excessive SUC and SAC intake during the juvenile period may involve both repeated strong pleasure (sweet taste) experience and the amount of caloric consumption.

In this study, we investigated the effects of SUC and SAC exposure in male animals. Although there may be some profits of using male mice, including controlling of subjects to minimize influence of various factors such as menstrual cycles, and enabling to compare with other studies investigating relevant issues in male animals, substance-related addiction has been reported more vulnerable to female than male subjects (Becker et al., 2017). Indeed, neurotransmitter mechanisms involved in addiction have been shown under tight relationships with sex hormones, such as estrogen and progesterone (Lightfoot, 2008). Thus, investigation with female animals remains a very important issue that was not addressed in the current study. Repeating all experiments conducted in the current study with female mice could double the amount of the data, and thereby, beyond the scope of the current study. However, we will investigate the effects of juvenile SUC and SAC exposure in female mice in our next study, with which we can compare the results with the current study conducted in male mice to elucidate if there are any gender differences.

Excessive consumption of SUC has been suggested as one of the major dietary factors associated with various chronic diseases, including obesity, diabetes, and cardiovascular disease (Howard and Wylie-Rosett, 2002; Tsilas et al., 2017; Burke et al., 2018; Togo et al., 2019). Thus, we expected to observe such augmented weight gain with repeated sucrose administration, but actually did not. As one of possible reasons, we suspect that these mice with sucrose administration compensated for their caloric intakes by eating less food pellets, so that they maintained body weight gain comparable to that of control mice. In addition, although chronic SUC consumption in adulthood has been shown to induce obesity with increasing body weight, the effects depend on the life-cycles, duration and amount of consumption, and genetic backgrounds of animals (Glendinning et al., 2010), such that excessive SUC intake only for 2 weeks in the juvenile period at the concentration of 20% solution might not be enough to induce apparent obesity. Recent studies suggested that excessive SUC consumption also induces brain dysfunctions, such as learning impairments and addiction-like behavior (Reichelt et al., 2015; Abbott et al., 2016; Wiss et al., 2018). Excessive SUC intake during pregnancy in rodents has also been reported to induce ADHD-like behavioral alterations with increased striatal DAT expression in offspring (Choi et al., 2015). Our study suggests that excessive SUC and SAC consumptions during the juvenile period induce an assortment of behavioral and neural alterations, some of which could further interact with maturation processes during adolescence, consequently causing both concurrent juvenile and adult expressions of alterations. Thus, excessive SUC consumption in early development may be a predisposition that increases the vulnerability of developing psychiatric disorders.

## Conclusion

Excessive intake of SUC in the juvenile period induced behavioral alterations caused by dysregulations of dopaminergic and serotonergic neuronal pathways both in juvenile and adult animals.

## Data Availability Statement

The datasets generated during and/or analyzed during the current study are available from the corresponding author on reasonable request.

## Ethics Statement

The animal study was reviewed and approved by the Institutional Animal Care and Use Committee of Daegu Catholic University.

## Author Contributions

Y-AL conceived the idea and designed the research. W-HC, K-AL, and YG performed the experiments. W-HC and K-AL contributed to data analyzation and sample preparation. Y-AL and YG wrote the manuscript. All authors contributed to the interpretation of the results, and provided critical feedback and helped shape the research, analysis and manuscript.

## Conflict of Interest

The authors declare that the research was conducted in the absence of any commercial or financial relationships that could be construed as a potential conflict of interest.

## References

[B1] Abbott K. N. Morris M. J. Westbrook R. F. Reichelt A. C. (2016). Sex-specific effects of daily exposure to sucrose on spatial memory performance in male and female rats, and implications for estrous cycle stage. *Physiol. Behav.* 162 52–60. 10.1016/j.physbeh.2016.01.036 26828038

[B2] Avena N. M. Rada P. Hoebel B. G. (2008). Evidence for sugar addiction: behavioral and neurochemical effects of intermittent, excessive sugar intake. *Neurosci. Biobehav. Rev.* 32 20–39. 10.1016/j.neubiorev.2007.04.019 17617461 PMC2235907

[B3] Bassareo V. Cucca F. Frau R. Di Chiara G. (2015). Differential activation of accumbens shell and core dopamine by sucrose reinforcement with nose poking and with lever pressing. *Behav. Brain Res.* 294 215–223. 10.1016/j.bbr.2015.08.006 26275926

[B4] Becker J. B. McClellan M. L. Reed B. G. (2017). Sex differences, gender and addiction. *J. Neurosci. Res.* 95 136–147. 10.1002/jnr.23963 27870394 PMC5120656

[B5] Best J. Nijhout H. F. Reed M. (2010). Serotonin synthesis, release and reuptake in terminals: a mathematical model. *Theor. Biol. Med. Model.* 7:34. 10.1186/1742-4682-7-34 PMC2942809 20723248

[B6] Bissonnette D. J. List S. Knoblich P. Hadley M. (2017). The effect of nonnutritive sweeteners added to a liquid diet on volume and caloric intake and weight gain in rats. *Obesity* 25 1556–1563. 10.1002/oby.21920 28763168

[B7] Burke S. J. Batdorf H. M. Martin T. M. Burk D. H. Noland R. C. Cooley C. R. (2018). Liquid sucrose consumption promotes obesity and impairs glucose tolerance without altering circulating insulin levels. *Obesity* 26 1188–1196. 10.1002/oby.22217 29901267 PMC6014929

[B8] Cathelain S. Brunault P. Ballon N. Réveillère C. Courtois R. (2016). Food addiction: Definition, measurement and limits of the concept, associated factors, therapeutic and clinical implications. *Presse Med.* 45 1154–1163. 27211587 10.1016/j.lpm.2016.03.014

[B9] Choi C. S. Kim P. Park J. H. Gonzales E. L. Kim K. C. Cho K. S. (2015). High sucrose consumption during pregnancy induced ADHD-like behavioral phenotypes in mice offspring. *J. Nutr. Biochem.* 26 1520–1526. 10.1016/j.jnutbio.2015.07.018 26452319

[B10] Cinque S. Zoratto F. Poleggi A. Leo D. Cerniglia L. Cimino S. (2018). Behavioral phenotyping of dopamine transporter knockout rats: compulsive traits, motor stereotypies, and anhedonia. *Front. Psychiatry* 9:43. 10.3389/fpsyt.2018.00043 29520239 PMC5826953

[B11] Erbaş O. Erdoğan M. A. Khalilnezhad A. Solmaz V. Gürkan F. T. Yiğittürk G. (2018). Evaluation of long-term effects of artificial sweeteners on rat brain: a biochemical, behavioral, and histological study. *J. Biochem. Mol. Toxicol.* 32:e22053. 10.1002/jbt.22053 29660801

[B12] Feijó F. M. Ballard C. R. Foletto K. C. Batista B. A. M. Neves A. M. Ribeiro M. F. M. (2013). Saccharin and aspartame, compared with sucrose, induce greater weight gain in adult Wistar rats, at similar total caloric intake levels. *Appetite* 60 203–207. 10.1016/j.appet.2012.10.009 23088901

[B13] Franklin K. B. Paxinos G. (2008). *The Mouse Brain In Stereotaxic Coordinates, Compact.* Amsterdam: Academic Press.

[B14] Geiger B. M. Behr G. G. Frank L. E. Caldera-Siu A. D. Beinfeld M. C. Kokkotou E. G. (2008). Evidence for defective mesolimbic dopamine exocytosis in obesity-prone rats. *FASEB J.* 22 2740–2746. 10.1096/fj.08-110759 18477764 PMC2728544

[B15] Geiger B. M. Haburcak M. Avena N. M. Moyer M. C. Hoebel B. G. Pothos E. N. (2009). Deficits of mesolimbic dopamine neurotransmission in rat dietary obesity. *Neuroscience* 159 1193–1199. 10.1016/j.neuroscience.2009.02.007 19409204 PMC2677693

[B16] Glendinning J. I. Breinager L. Kyrillou E. Lacuna K. Rocha R. Sclafani A. (2010). Differential effects of sucrose and fructose on dietary obesity in four mouse strains. *Physiol. Behav.* 101 331–343. 10.1016/j.physbeh.2010.06.003 20600198 PMC2930118

[B17] Gould T. J. (2010). Addiction and cognition. *Addict. Sci. Clin. Pract.* 5 4–14. 22002448 PMC3120118

[B18] Hajnal A. Smith G. P. Norgren R. (2004). Oral sucrose stimulation increases accumbens dopamine in the rat. *Am. J. Physiol. Regul. Integr. Comp. Physiol.* 286 R31–R37. 12933362 10.1152/ajpregu.00282.2003

[B19] Hebebrand J. Albayrak O. Adan R. Antel J. Dieguez C. de Jong J. (2014). Eating addiction, rather than “food addiction”, better captures addictive-like eating behavior. *Neurosci. Biobehav. Rev.* 47 295–306. 10.1016/j.neubiorev.2014.08.016 25205078

[B20] Howard B. V. Wylie-Rosett J. (2002). Sugar and cardiovascular disease: a statement for healthcare professionals from the committee on nutrition of the council on nutrition, physical activity, and metabolism of the american heart association. *Circulation* 106 523–527. 10.1161/01.cir.0000019552.77778.04 12135957

[B21] Innos J. Philips M. A. Leidmaa E. Heinla I. Raud S. Reemann P. (2011). Lower anxiety and a decrease in agonistic behaviour in Lsamp-deficient mice. *Behav. Brain Res.* 217 21–31. 10.1016/j.bbr.2010.09.019 20888367

[B22] Jeon S. Y. Kim N. H. Kim Y. J. Lee K. A. Goto Y. Lee Y. A. (2019). The effects of Engelhardtia chrysolepis hance on long-term memory and potential dopamine involvement in mice. *Behav. Pharmacol.* 30 596–604. 10.1097/fbp.0000000000000495 31503068

[B23] Kim Y. J. Goto Y. Lee Y. A. (2018). Histamine H3 receptor antagonists ameliorate attention deficit/hyperactivity disorder-like behavioral changes caused by neonatal habenula lesion. *Behav. Pharmacol.* 29 71–78. 10.1097/fbp.0000000000000343 28863002

[B24] Kimmel H. L. Carroll F. I. Kuhar M. J. (2003). Withdrawal from repeated cocaine alters dopamine transporter protein turnover in the rat striatum. *J. Pharmacol. Exp. Ther.* 304 15–21. 10.1124/jpet.102.038018 12490570

[B25] Kranz G. S. Kasper S. Lanzenberger R. (2010). Reward and the serotonergic system. *Neuroscience* 166 1023–1035. 10.1016/j.neuroscience.2010.01.036 20109531

[B26] Lee Y. A. Obora T. Bondonny L. Toniolo A. Mivielle J. Yamaguchi Y. (2018). The Effects of housing density on social interactions and their correlations with serotonin in rodents and primates. *Sci. Rep.* 8:3497. 10.1038/s41598-018-21353-6 PMC5823940 29472615

[B27] Lenoir M. Serre F. Cantin L. Ahmed S. H. (2007). Intense sweetness surpasses cocaine reward. *PLoS One* 2:e698. 10.1371/journal.pone.0000698 17668074 PMC1931610

[B28] Lightfoot J. T. (2008). Sex hormones’ regulation of rodent physical activity: a review. *Int. J. Biol. Sci.* 4 126–132. 10.7150/ijbs.4.126 18449357 PMC2359866

[B29] Lindseth G. N. Coolahan S. E. Petros T. V. Lindseth P. D. (2014). Neurobehavioral effects of aspartame consumption. *Res. Nurs. Health* 37 185–193. 10.1002/nur.21595 24700203 PMC5617129

[B30] Meiser J. Weindl D. Hiller K. (2013). Complexity of dopamine metabolism. *Cell. Commun. Signal.* 11:34. 10.1186/1478-811x-11-34 23683503 PMC3693914

[B31] Meule A. (2015). Back by popular demand: a narrative review on the history of food addiction research. *Yale J. Biol. Med.* 88 295–302. 26339213 PMC4553650

[B32] Meule A. Gearhardt A. N. (2014). Food addiction in the light of DSM-5. *Nutrients* 16 3653–3671. 10.3390/nu6093653 25230209 PMC4179181

[B33] Müller C. P. Homberg J. R. (2015). The role of serotonin in drug use and addiction. *Behav. Brain Res.* 277 146–192. 10.1016/j.bbr.2014.04.007 24769172

[B34] Murphy C. M. Stojek M. K. Mackillop J. (2014). Interrelationships among impulsive personality traits, food addiction, and body mass index. *Appetite* 73 45–50. 10.1016/j.appet.2013.10.008 24511618 PMC4859335

[B35] Murray S. Tulloch A. Criscitelli K. Avena N. M. (2016). Recent studies of the effects of sugars on brain systems involved in energy balance and reward: relevance to low calorie sweeteners. *Physiol. Behav.* 164 504–508. 10.1016/j.physbeh.2016.04.004 27068180 PMC5003688

[B36] Nissbrandt H. Carlsson A. (1987). Turnover of dopamine and dopamine metabolites in rat brain: comparison between striatum and substantia nigra. *J. Neurochem.* 49 959–967. 10.1111/j.1471-4159.1987.tb00987.x 3612134

[B37] Quello S. B. Brady K. T. Sonne S. C. (2005). Mood disorders and substance use disorder: a complex comorbidity. *Sci. Pract. Perspect.* 3 13–21. 10.1151/spp053113 18552741 PMC2851027

[B38] Randolph T. G. (1956). The descriptive features of food addiction; addictive eating and drinking. *Q. J. Stud. Alcohol.* 17 198–224. 10.15288/qjsa.1956.17.198 13336254

[B39] Reichelt A. C. Killcross S. Hambly L. D. Morris M. J. Westbrook R. F. (2015). Impact of adolescent sucrose access on cognitive control, recognition memory, and parvalbumin immunoreactivity. *Learn. Mem.* 22 215–224. 10.1101/lm.038000.114 25776039 PMC4371171

[B40] Robinson T. E. Kolb B. (2004). Structural plasticity associated with exposure to drugs of abuse. *Neuropharmacology* 47 33–46. 10.1016/j.neuropharm.2004.06.025 15464124

[B41] Salahpour A. Ramsey A. J. Medvedev I. O. Kile B. Sotnikova T. D. Holmstrand E. (2008). Increased amphetamine-induced hyperactivity and reward in mice overexpressing the dopamine transporter. *Proc. Natl. Acad. Sci. U.S.A.* 105 4405–4410. 10.1073/pnas.0707646105 18347339 PMC2393812

[B42] Smeets P. A. Weijzen P. de Graaf C. Viergever M. A. (2011). Consumption of caloric and non-caloric versions of a soft drink differentially affects brain activation during tasting. *Neuroimage* 54 1367–1374. 10.1016/j.neuroimage.2010.08.054 20804848

[B43] Smith J. E. Koves T. R. Co C. (2003). Brain neurotransmitter turnover rates during rat intravenous cocaine self-administration. *Neuroscience* 117 461–475. 10.1016/s0306-4522(02)00819-9 12614686

[B44] Smolders I. Loo J. V. Sarre S. Ebinger G. Michotte Y. (2001). Effects of dietary sucrose on hippocampal serotonin release: a microdialysis study in the freely-moving rat. *Br. J. Nutr.* 86 151–155. 10.1079/bjn2001360 11502227

[B45] Togo J. Hu S. Li M. Niu C. Speakman J. R. (2019). Impact of dietary sucrose on adiposity and glucose homeostasis in C57BL/6J mice depends on mode of ingestion: liquid or solid. *Mol. Metab.* 27 22–32. 10.1016/j.molmet.2019.05.010 31255519 PMC6717800

[B46] Tsilas C. S. de Souza R. J. Mejia S. B. Mirrahimi A. Cozma A. I. Jayalath V. H. (2017). Relation of total sugars, fructose and sucrose with incident type 2 diabetes: a systematic review and meta-analysis of prospective cohort studies. *CMAJ* 189 E711–E720. 28536126 10.1503/cmaj.160706 PMC5436961

[B47] Volkow N. D. Wang G. J. Fowler J. S. Tomasi D. Telang F. (2011). Addiction: beyond dopamine reward circuitry. *Proc. Natl. Acad. Sci. U.S.A.* 108 15037–15042. 21402948 10.1073/pnas.1010654108 PMC3174598

[B48] Walf A. A. Frye C. A. (2007). The use of the elevated plus maze as an assay of anxiety-related behavior in rodents. *Nat. Protoc.* 2 322–328. 10.1038/nprot.2007.44 17406592 PMC3623971

[B49] Wang G. J. Volkow N. D. Logan J. Pappas N. R. Wong C. T. Zhu W. (2001). Brain dopamine and obesity. *Lancet* 357 354–357. 11210998 10.1016/s0140-6736(00)03643-6

[B50] Wiss D. A. Avena N. Rada P. (2018). Sugar Addiction: From Evolution to Revolution. *Front. Psychiatry* 9:545 10.3389/fpsyt.2018.000545 PMC6234835 30464748

[B51] Yankelevitch-Yahav R. Franko M. Huly A. Doron R. (2015). The forced swim test as a model of depressive-like behavior. *J. Vis. Exp.* 97:52587. 10.3791/52587 PMC4401172 25867960

