# Supplementary material for: Concurrent and Delayed Behavioral and Monoamine Alterations by Excessive Sucrose Intake in Juvenile Mice
Source: Front Neurosci. 2020 May 19;14:504. doi: 10.3389/fnins.2020.00504 (PMC7248345; doi:10.3389/fnins.2020.00504)
Supplement: Supplementary file 1 [file Table_1.pdf]

Supplementary Information

Concurrent and Delayed Behavioural and Monoamine Alterations
by Excessive Sucrose Intake in Juvenile Mice

Won-Hui Choe, Kyung-A Lee, Yukiori Goto, and Young-A Lee

Supplementary Table S1

Supplementary Table S2

Supplementary Table S3

Supplementary Figure S1

Supplementary Table S1. Summary of behavioral alterations induced by excessive SUC and SAC intakes in the juvenile period.

	Juvenile		Adulthood	
	**SUC**	**SAC**	**SUC**	**SAC**
**AMP Response**	---	Lower at 3 mg/kg	Stronger	Weaker
**Sucrose Preference**	Lower at 5%	Lower at 5%	---	---
**Stress Response**	Higher	---	---	---
**Anxiety**	---	Lower	Higher	---
**Attention**	---	---	---	---
**Social Behavior**	---	---	---	---

Supplementary Table S2. Summary of monoamine alterations in juvenile animals induced by excessive SUC and SAC intakes in the juvenile period.

	DA		DOPAC/DA		HVA/DA		DAT			5-HT		5-HIAA/5-HT		SERT	
	SUC	SAC	SUC	SAC	SUC	SAC	SUC	SAC		SUC	SAC	SUC	SAC	SUC	SAC
**PFC**	---	---	---	---	---	---	---	---	**PFC**	---	---	---	---	---	---
**dSTR**	Low	Low	---	High	---	High	---	---	**dSTR**	Low	Low	---	High	High	---
**NAcc**	---	Low	---	High	---	High	---	---	**NAcc**	---	Low	---	High	---	---
**HPC**	---	---	---	---	---	---	---	---	**HPC**	---	---	---	---	---	---
**AMY**	---	---	---	---	---	---	---	---	**AMY**	---	---	---	---	---	---
**VTA**	---	---	---	---	---	---	---	---	**VTA**	---	---	---	---	---	---

Supplementary Table S3. Summary of monoamine alterations in adult animals induced by excessive SUC and SAC intakes in the juvenile period.

	DA		DOPAC/DA		HVA/DA		DAT			5-HT		5-HIAA/5-HT		SERT	
	**SUC**	**SAC**	**SUC**	**SAC**	**SUC**	**SAC**	**SUC**	**SAC**		**SUC**	**SAC**	**SUC**	**SAC**	**SUC**	**SAC**
**PFC**	---	---	---	---	---	---	High	High	**PFC**	---	---	---	---	High	High
**dSTR**	---	---	---	---	---	---	High	High	**dSTR**	---	---	---	---	High	High
**NAcc**	High	---	---	---	---	---	High	---	**NAcc**	---	---	---	---	High	High
**HPC**	---	High	---	---	---	---	---	---	**HPC**	---	---	---	---	---	High
**AMY**	Low	Low	---	---	---	---	High	---	**AMY**	---	---	Low	Low	High	High
**VTA**	High	High	Low	Low	---	---	---	---	**VTA**	---	---	Low	Low	---	---

Supplementary Figure S1. Control images for the fluorescence expression of brain tissue by Alexa Fluor® 488 andAlexa Fluor® 594antibodies

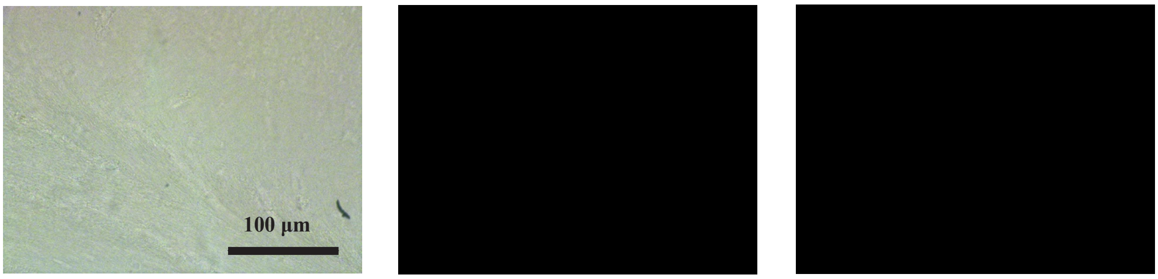

BF filter 488nmfilter 594 nmfilter
